# Polycrystalline texture causes magnetic instability in greigite

**DOI:** 10.1038/s41598-020-80801-4

**Published:** 2021-02-04

**Authors:** Barbara Lesniak, Dimitrios Koulialias, Michalis Charilaou, Peter G. Weidler, Jordan M. Rhodes, Janet E. Macdonald, Andreas U. Gehring

**Affiliations:** 1 Department of Earth Sciences, Institute of Geophysics, ETH Zurich, 8092 Zurich, Switzerland; 2 Department of Physics, University of Louisiana at Lafayette, Lafayette, LA 70504 USA; 3 Institute of Functional Interfaces, Karlsruhe Institute of Technology, 76021 Karlsruhe, Germany; 4 Department of Chemistry, Vanderbilt University and Vanderbilt Institute for Nanoscale Science and Engineering, Nashville, TN 37235 USA

**Keywords:** Magnetic properties and materials, Mineralogy, Palaeomagnetism

## Abstract

Magnetic stability of iron mineral phases is a key for their use as paleomagnetic information carrier and their applications in nanotechnology, and it critically depends on the size of the particles and their texture. Ferrimagnetic greigite (Fe_3_S_4_) in nature and synthesized in the laboratory forms almost exclusively polycrystalline particles. Textural effects of inter-grown, nano-sized crystallites on the macroscopic magnetization remain unresolved because their experimental detection is challenging. Here, we use ferromagnetic resonance (FMR) spectroscopy and static magnetization measurements in concert with micromagnetic simulations to detect and explain textural effects on the magnetic stability in synthetic, polycrystalline greigite flakes. We demonstrate that these effects stem from inter-grown crystallites with mean coherence length (MCL) of about 20 nm in single-domain magnetic state, which generate modifiable coherent magnetization volume (CMV) configurations in the flakes. At room temperature, the instability of the CVM configuration is exhibited by the angular dependence of the FMR spectra in fields of less than 100 mT and its reset by stronger fields. This finding highlights the magnetic manipulation of polycrystalline greigite, which is a novel trait to detect this mineral phase in Earth systems and to assess its fidelity as paleomagnetic information carrier. Additionally, our magneto-spectroscopic approach to analyse instable CMV opens the door for a new more rigorous magnetic assessment and interpretation of polycrystalline nano-materials.

## Introduction

Greigite (Fe_3_S_4_) is a ferrimagnetic thio-spinel isomorphic to magnetite, the archetypical mineral in natural magnetism. Since the discovery of greigite in Miocene lake sediments by Skinner et al.^1^, it has been widely used as powerful sulfidic proxy for geochemical reconstructions and as a remanence carrier to record paleomagnetic information^2–6^. Fe_3_S_4_ nano-particles have also attracted considerable attention owing to the morphology, and the magnetic and electronic properties^7–11^ for technical applications in areas such as spintronics^12^, catalysis^13^, and biomedicine^14^. Greigite in nature generally forms nano-sized crystallites with variable morphologies, which can aggregate in clumps and nodules^5,15–17^, and similar to magnetite, nano-sized greigite can also form intracellular chain assemblies in magnetotactic bacteria^18–20^. Laboratory studies showed that the morphology of greigite nanoparticles and the formation of assemblies can be tailored by the synthesis process, e.g., by the selection of the organosulfur precursor^21,22^ and that the architecture of the assemblies influences the magnetic properties^23^. In contrast to the oxide counterpart magnetite, the fundamental magnetic properties and especially its size dependence in the nano-meter scale have not been conclusively resolved for greigite and this may limit numerical approaches to infer the physical characteristics^9,12,24–26^. For pure synthetic, μm-sized, cubo-octahedreal crystals with a saturation magnetization *M*_s_ ≈ 60 Am^2^ kg^−1^ was reported whereas the Curie temperature (*T*_c_) remained ill-defined due to thermal phase instability^5,26,27^. Ferromagnetic resonance (FMR) spectroscopy was applied to constrain the magnetocrystalline anisotropy constants^24^. These reported magnetic parameters were obtained from polycrystalline particles, and importantly, the coalescence effects of crystallites were not considered^5,27,28^. Such effect may generate magnetization textures with competitive single crystallite anisotropy properties and their interactions can critically affect the size and stability of coherent magnetic volumes (CMV)^29,30^. The impact of nano-texturization on the magnetic properties is vital to assess the fidelity of greigite for paleomagnetic studies of the Earth’s history and the use of the polycrystalline greigite for recording or technical applications. Here, we present a magneto-spectroscopic approach that detects CMV changes in polycrystalline greigite flakes, providing a new perspective to decipher the magnetic stability of nano-textured Earth materials.

## Results and discussion

### Structural properties

**Figure 1:**
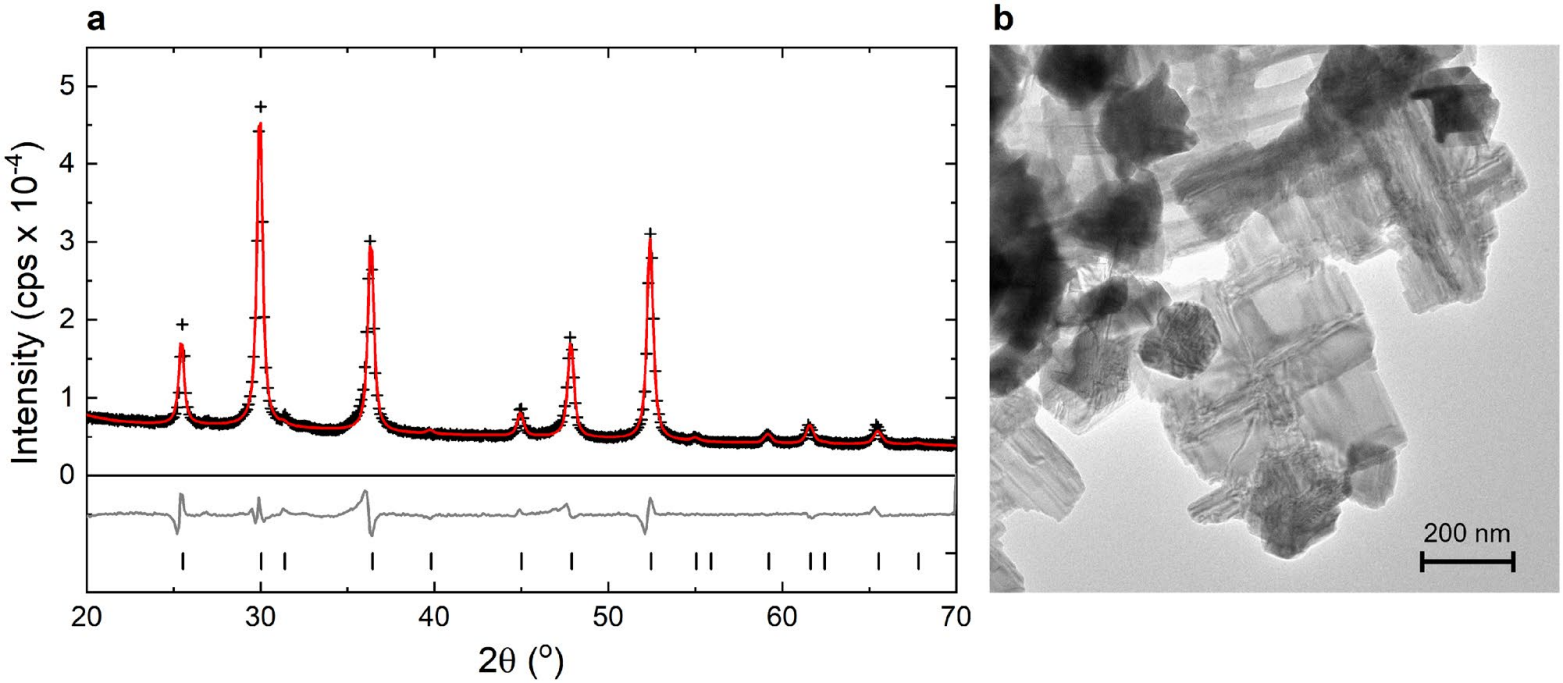
Characterisation of the synthesized greigite sample. (**a**) powder X-ray diffractogram with measured data point (+), fitted data (red line) and the difference between measurement and model (grey line). (**b**) TEM picture showing the flake morphology of the greigite.

X-ray diffractometry (XRD) exhibits peaks that can be exclusively attributed to greigite, i.e., the synthesized sample is a pure mineral single-phase (Fig. 1a). Moreover, the background of the XRD pattern clearly indicates that the amount of X-ray amorphous constituents is negligible. The Rietveld refinement converged with a goodness of fit of 4.53 and the R_Bragg_ of 1.007. The obtained lattice parameter *a* = 0.98580 ± 0.00016 nm is in excellent agreement with the literature values for greigite^1^. In addition, the mean coherence length (MCL) is 19 ± 2 nm and the stress/strain parameter *ε*_o_ is low with 0.00002 for our sample. Transmission electron microscopy (TEM) of the synthesized greigite powder reveals agglomerated nanoparticles in a size range generally between 100 and 200 nm with rectangular flake morphologies (Fig. 1b). This size range and the MCL inferred from XRD indicate polycrystalline flakes with textures formed by intergrown nano-crystallites. Such polycrystalline composite was reported for nano-sized^31^ and larger greigite particles^5^. Furthermore, the absence of significant strain/stress suggests that the polycrystallinity of the flakes most likely stems from crystal growth and is not a mechanical effect. With this in mind polycrystalline flakes can be considered as the result of the intergrowth of crystallites along variable principle crystallographic axes.

### Magnetic properties

**Figure 2:**
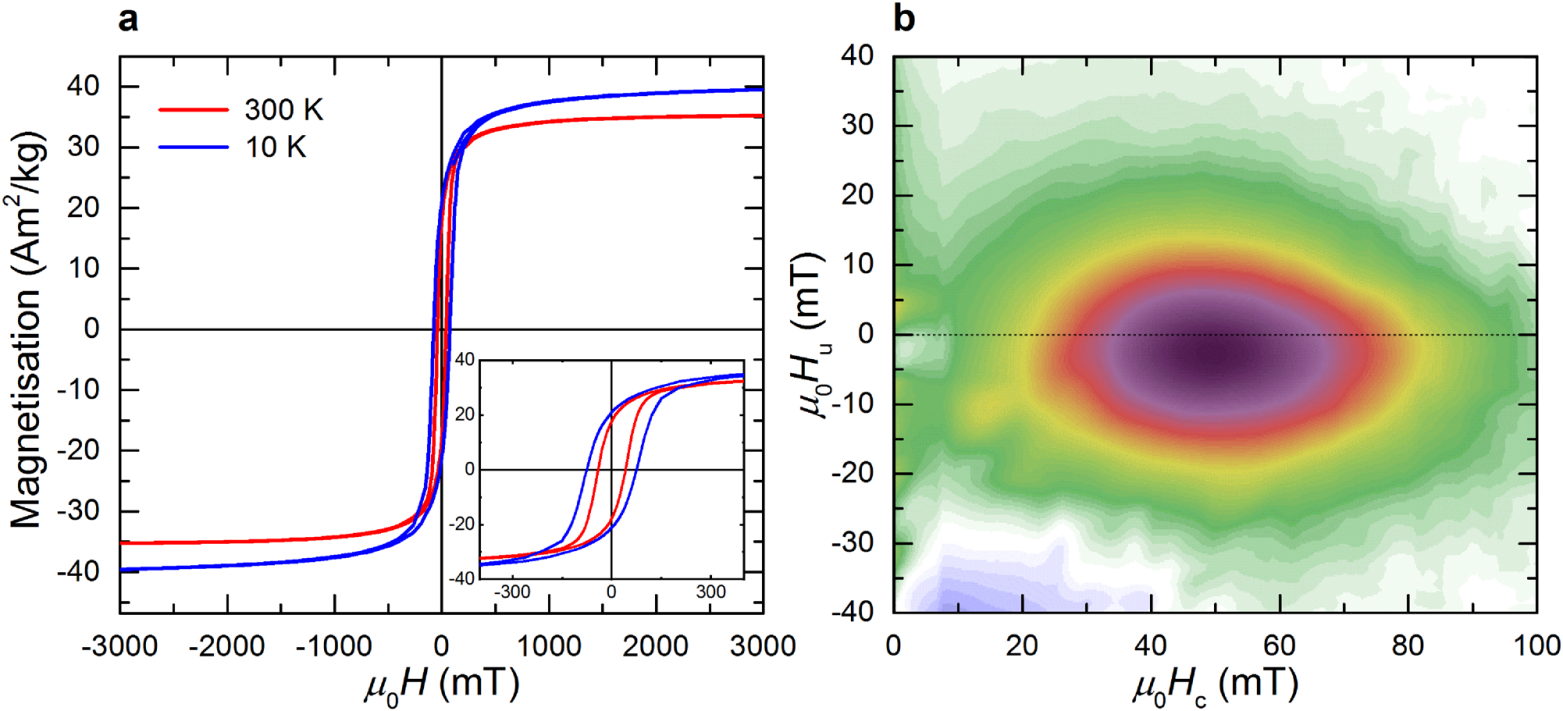
Magnetic hysteresis properties. (**a**) *M*(*H*) plots at 300 K and 10 K and enlarged hysteresis loops (inset). (**b**) FORC diagram obtained by processing of magnetic data recorded in fields up to 1 T, SF = 8; with nearly symmetric *H*_c_ distribution at *H*_u_ = 0 with highest values in dark purple.

At room temperature, the hysteresis loop in a 3 T field of the polycrystalline flakes shows *M*_3T_ = 34.2 Am^2^ kg^1^, a remanence *M*_r_ = 18.4 Am^2^ kg^−1^, *M*_r_/*M*_1T_ = 0.54, and a coercivity *μ*_0_*H*_*c*_ = 38.2 mT (Fig. 2a). In a 1 T field the hysteresis parameters are nearly the same with *M*_1T_ = 32.7 Am^2^ kg^−1^, *M*_r_ = 17.7 Am^2^ kg^−1^, a coercivity *μ*_0_*H*_*c*_ = 41.2 mT and a coercivity of remanence *μ*_0_*H*_*cr*_ = 54.8 mT. The squareness ratio *M*_r_/*M*_1T_ = 0.54 and *H*_*cr*_/*H*_*c*_ = 1.33 are indicative of uniaxial single domain (SD) particles^32^. The corresponding first order reversal curve (FORC) contour plot has an oval shape with a nearly symmetric *μ*_0_*H*_*c*_ distribution in the range of 10 and 100 mT with a maximum at about 50 mT that is in accordance with SD greigite (Fig. 2b). Moreover, the contour plot is shifted to the negative internal bias field (*H*_u_), which has been interpreted as a magnetostatic interaction effect^32^. It is worth noting that Hu et al.^5^ previously mentioned that the SD magnetic state of greigite particles in the 100 nm order of magnitude stems from their polycrystalline texture with nano-sized MCL. The thresholds of the magnetic domain states for greigite are poorly constrained. Micromagnetic modelling, however, suggests that greigite crystallites with MCL of about 20 nm are likely in SD magnetic state and that their pseudo-single domain/multidomain (PSD/MD) threshold is about 200 nm^11^. Given this, the magnetization of polycrystalline flakes with sizes of about 200 nm (Fig. 1b) can be considered as the sum of the magnetizations of intergrown SD crystallites.

The saturation magnetization (*M*_s_) of greigite reported in the literature varies and the highest value *M*_s_ ≈ 60 Am^2^ kg^−1^ was found for µm-sized, cubo-octahedral crystals^8,26^. Synthetic and natural nano-sized greigite reveal much lower apparent *M*_s_ of generally less than 40 Am^2^ kg^−1^^28^. Considering the XRD pattern and its background, it is very unlikely that the relatively low value *M*_1T_ = 32.7 Am^2^ kg^−1^ of our sample is due to an additional sulfide phase or X-ray amorphous constituents. Therefore, the departure from the expected *M*_s_ is most probably caused by inhomogeneities, i.e., at the surfaces and interfaces of the crystalites^33^. It is worth noting that a similar observation was reported for polycrystalline greigite nanoparticles with MCL of 20 nm^28^.

Low-temperature measurements exhibit continuous development of the hysteresis parameters with *M*_3T_ = 37.5 Am^2^ kg^−1^ and *μ*_0_*H*_*c*_ = 75 mT at 10 K, but the corresponding change in *M*_r_/*M*_3T_ is marginal (Fig. 2a). A similar *H*_*c*_ behaviour was reported by Coey et al*.*^34^ for a nano-sized polycrystalline greigite powder. Such behaviour and the nearly constant *M*_r_/*M*_3T_ ratio confirm that greigite has no low-temperature anomaly as it is known for the isomorphic magnetite with its Verwey transition^27,34^.

**Figure 3:**
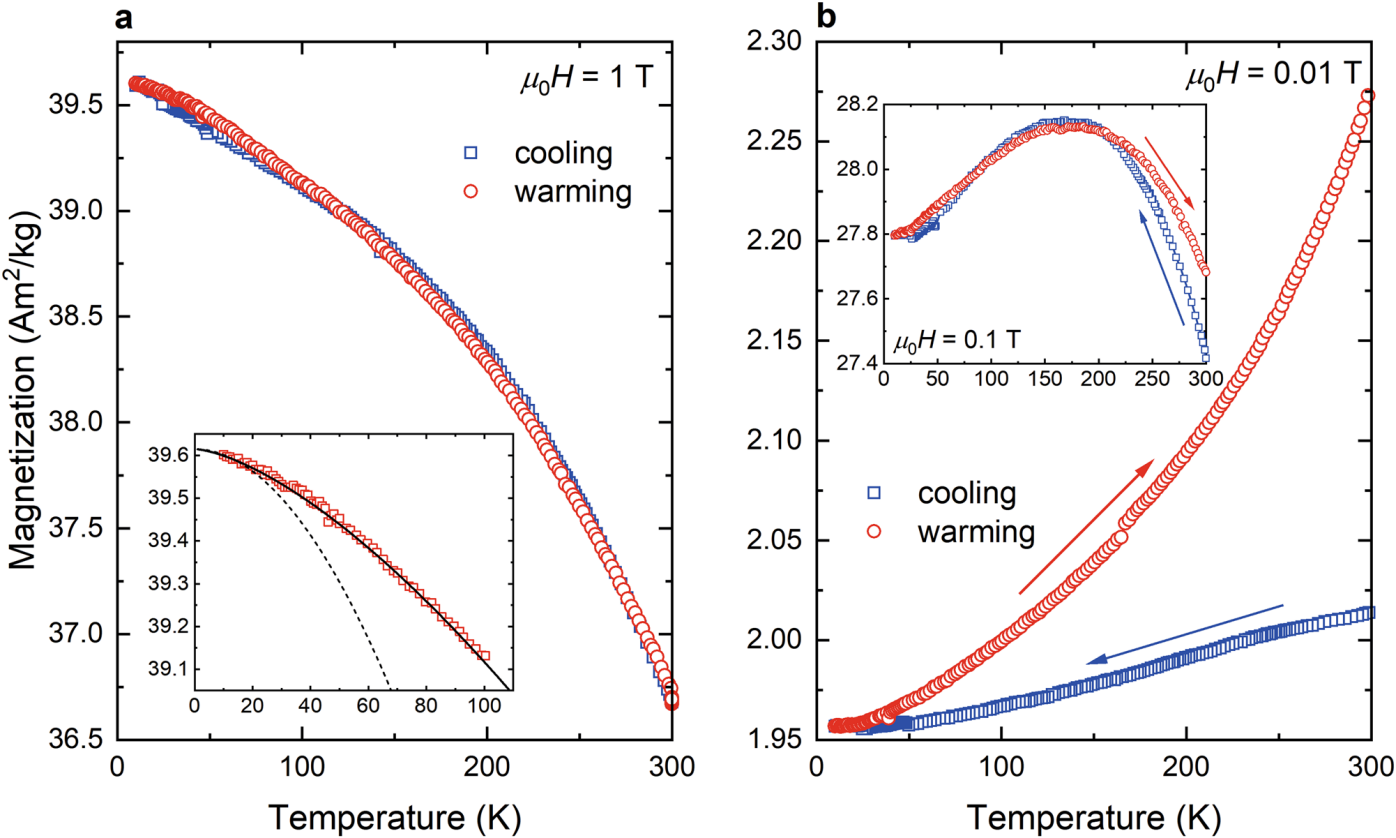
Low-temperature magnetization cycling. (**a**) Curves recorded in a 1 T field with inset shows the fit using the Bloch law with non-constraining *T*_c_ (solid line), dashed line with *T*_c_ = 600 K (see text). (**b**) curves recorded in a 10 mT field and a 100 mT field (inset).

Magnetization cycling experiments at low temperature reveal field-dependent behaviour (Fig. 3). Congruent cooling and warming curves were found for *μ*_0_*H* ≥ 0.5 T (Fig. 3a). In a field of 1 T that apparently saturates the bulk of the greigite crystallites (Fig. 2a), the curves can be fitted with the Bloch law *M*(*T*) = *M*_0_ (1 − (*T/T*_*c*_)^3/2^), where *M*_0_ is the magnetization at *T* = 0 and *T*_c_ is the Curie temperature (Fig. 3a). Considering the estimated *T*_c_ ≈ 600 K for greigite^26^ *M* follows the *T*^3/2^ law only in a temperature range between 5 and 20 K (Fig. 3a inset). Fitting over a wider temperature range results in unrealistic *T*_c_ > 1000 K. Chang et al.^26^ reported a similar behaviour for synthetic, μm-sized greigite particles with *M*_s_ ≈ 60 Am^2^ kg^1^. The cycling experiment with *μ*_0_*H* = 0.5 T, however, also reveals reversible magnetic behaviour of the cooling and warming curves. Irreversibility occurs in magnetic fields between 10 and 100 mT, which correspond to the coercivity range found in the FORC diagram (Figs. 2b, 3b). This behaviour is relatively weak in 100 mT, where the cycling increases the magnetization at 300 K (Δ*M*_300K_) by about 1% (Fig. 3b, inset). Both curves exhibit a concave shape with a maximum at *T* ≈ 160 K. Such behaviour is known for pyrrhotite (Fe_7_S_8_) and was explained by the interplay between the Zeeman energy (*E*_*z*_) and the magnetocrystalline anisotropy energy^35^. For the greigite flakes the departure from the Bloch law, however, can be attributed to the absence of saturation and to the finite size of the crystallites and their interfaces, which affect the local chemical potential^36,37^. To test this interpretation, the cycling experiments were performed in weaker fields, under the assumption that crystallite and interface effects become more pronounced by lowering *E*_*z*_. In a field *μ*_0_*H* = 10 mT, the cooling curve exhibits a nearly linear decrease of about 3% down to 10 K and a subsequent monotonic increase upon warming that follows a power law (Fig. 3b). After the cycling, the gain in magnetization at 300 K (Δ*M*_300K_) is 12.9%. In general, SD particles exhibit similar cooling and warming curves whereas MD particles show demagnetization during the cycling due to domain wall dynamics^35,38^. Amplitude-dependent *ac* susceptibility up to *μ*_0_*H*_ac_ ≤ 1.5 mT at 300 K provides no evidence of domain-wall motions in our polycrystalline greigite flakes, i.e., the domain walls associated with the interfaces are relatively stiff. Given this, the peculiar behaviour of the flakes is probably an extrinsic, textural effect. Studies on nanoparticle ensembles showed that competition between crystalllites can critically affect the CMV of the constituents^29^ and consequently the magnetization texture of a sample. Considering the MCL of 19 ± 2 nm for our flakes, the Δ*M*_300K_ shift to zero with increasing field points to polycrystalline textures with changing CMV. In a near saturation field where Δ*M*_300K_ = 0% the different CMV configurations in the flakes are wiped out. The irreversible magnetization during thermal cycling in *μ*_0_*H* ≤ 100 mT implies that a weak field modifies the CMV and this in turn the magnetization texture of the flakes.

### FMR spectroscopy

**Figure 4:**
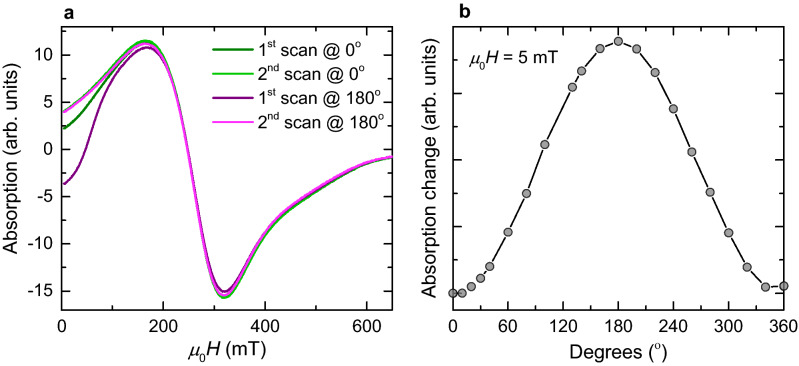
Rotational behaviour of FMR spectra at 300 K. (**a**) Initial and second spectra in 0° positions and first and second spectra in 180° positions with varying absorption behaviours in the low-field range at *μ*_0_*H* < 200 mT and with second scans superimposed. (**b**) Angular effect of the absorption in *μ*_0_*H* = 5 mT depicted as the departure from the reference absorption [2nd scans shown in (**a**)] in absolute values.

FMR spectroscopy can be used to experimentally constrain the interplay between anisotropy and magnetostatic interactions. The spectrum of the polycrystalline flakes at 300 K is asymmetric with a symmetry ratio *A*_*ratio*_ = 0.84, i.e., negative skewness^39^, and with spectral parameters *μ*_0_*H*_*res*_ = 247.6 mT, *g* = 2.85 and *μ*_0_Δ*H* = 155 mT and it exhibits near zero-field absorption (Fig. 4a). The spectra obtained from dispersed powder samples of synthetic, μm-sized PSD/MD greigite particles reported by Chang et al.^40^ show quite similar spectral parameters but positive skewness (*g* = 2.95–3.13, *μ*_0_ Δ*H* ≈ 200 mT, and *A*_*ratio*_ > 1). This implies that grain-size and shape of polycrystalline particles have only minor effect on the spectral parameters at 300 K. The different skewness of the spectra most likely stems from the sign of the magnetocrystalline anisotropy constants and/or the orientation of the easy axes^24^. Pronounced near-zero field absorption has been generally reported for relatively large MD particles due to domain wall effects^41,42^. Such effects are unexpected for the greigite flakes, which show a magnetic response in the hysteresis measurements typical of SD state (Fig. 2a,b). Therefore, the near-zero field absorption most likely stems from interfaces between nano-crystallites. The FMR spectra at 300 K, however, reveal angular dependence of the absorption intensity in the range of *μ*_0_*H* < 200 mT and no angular dependence occurs at higher fields as it is generally expected for powder samples (Fig. 4a). In fields less than 200 mT, where greigite flakes are non-saturated (Fig. 2a), the absorption behaviour changes with the external field orientation and the directional differences tend to zero with increasing fields (Fig. 4a). Moreover, the microwave absorption reveals 2π rotational dependence in a low-field range where the configurational entropy is relatively high (Fig. 4b). This points to induced magnetization of the CMV configurations, which are affected by the geometry of the intergrown nano-crystallites, i.e., the angle between their easy axes and the external field. Considering the above, the angular dependence provides clear evidence that in the low-field range, the randomization of the flakes in the sample is broken with respect to the CMV of the polycrystalline matrix. This breaking is annihilated by remagnetization in a sweeping field up to 0.65 T and the initial FMR response is re-established, i.e., the CMV of the flake exhibits a memory effect.

**Figure 5:**
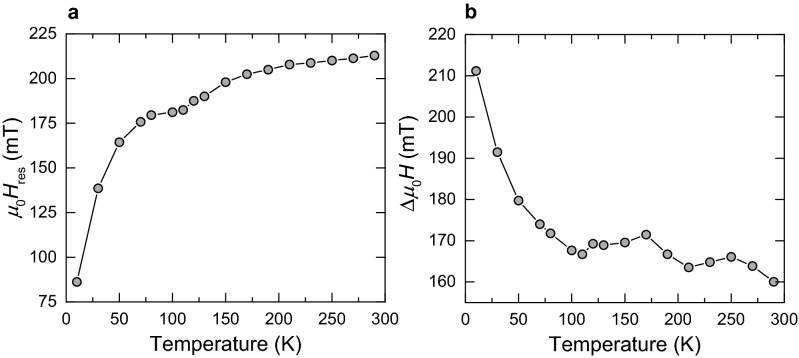
Development of the spectral parameters at low temperatures. (**a**) *μ*_0_*H*_res_ and (**b**) Δ*μ*_0_*H* showing pronounced changes at *T* $\approx$ 50 K.

Figure 5 shows the change of the spectral parameters upon cooling. The resonance field-strength continuously declines down to about 50 K followed by a prominent decrease and the corresponding increase of the *g*-value to 7.78 at 10 K. A pronounced decrease in *H*_*res*_ is well known for magnetite at the Verwey transition^43^, which is associated with changes of the crystallographic structure from cubic to monoclinic and of the magnetocrystalline anisotropy as documented by a shift of the easy axes from (111) to (100). For such transition in greigite, there is no indication in our magnetization curves (Fig. 3) and also no report in the literature^27,34^. Associated with the pronounced change of *H*_*res*_, Δ*H* markedly increases without significant change in *A*_ratio_. The linewidth broadening points to pronounced increase in interaction energy of the crystallites within the flakes and the simultaneous shift in the *g*-value can be explained by changes in CMV and their anisotropy properties.

**Figure 6:**
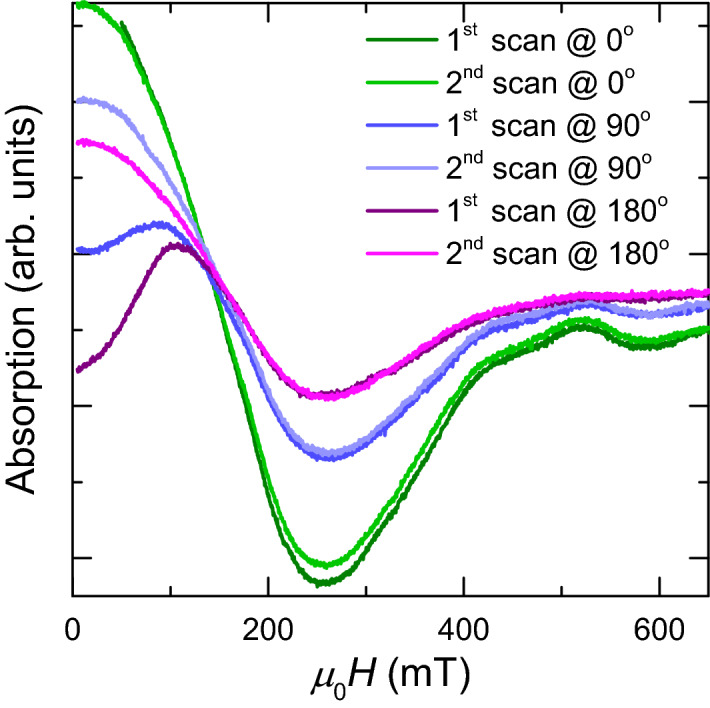
Angular dependence of the FMR spectra at 10 K. First and second scans of the initial spectrum and after rotations by 90° and 180°, respectively.

The broad spectra recorded at 10 K show angular dependence that is manifested over the whole sweeping field range (Fig. 6). For the high-field absorption at *μ*_0_*H* > 150 mT mainly the signal intensity varies. For the low-field range *μ*_0_*H* < 150 mT the absorptions conspicuously change without a clear angular pattern (Fig. 6). Repeated scans under constant angles generate low-field absorptions that exhibit decreasing intensity with increasing angle between the external field and the sample (Fig. 6). For the high-field absorptions the sweeping field up to 0.65 T has nearly no effect. Compared to 300 K, the spectral properties at 10 K imply that at low-temperature stable, inerasable CMV configurations are induced. Considering the low-temperature behaviour of the spectral parameters (Fig. 5), it can be supposed that this effect is more pronounced at *T* < 50 K. Furthermore, changes in the CMV configurations could explain the peculiar temperature dependence of the magnetization in the cycling experiments (Fig. 3b).

### Micromagnetic simulations

**Figure 7:**
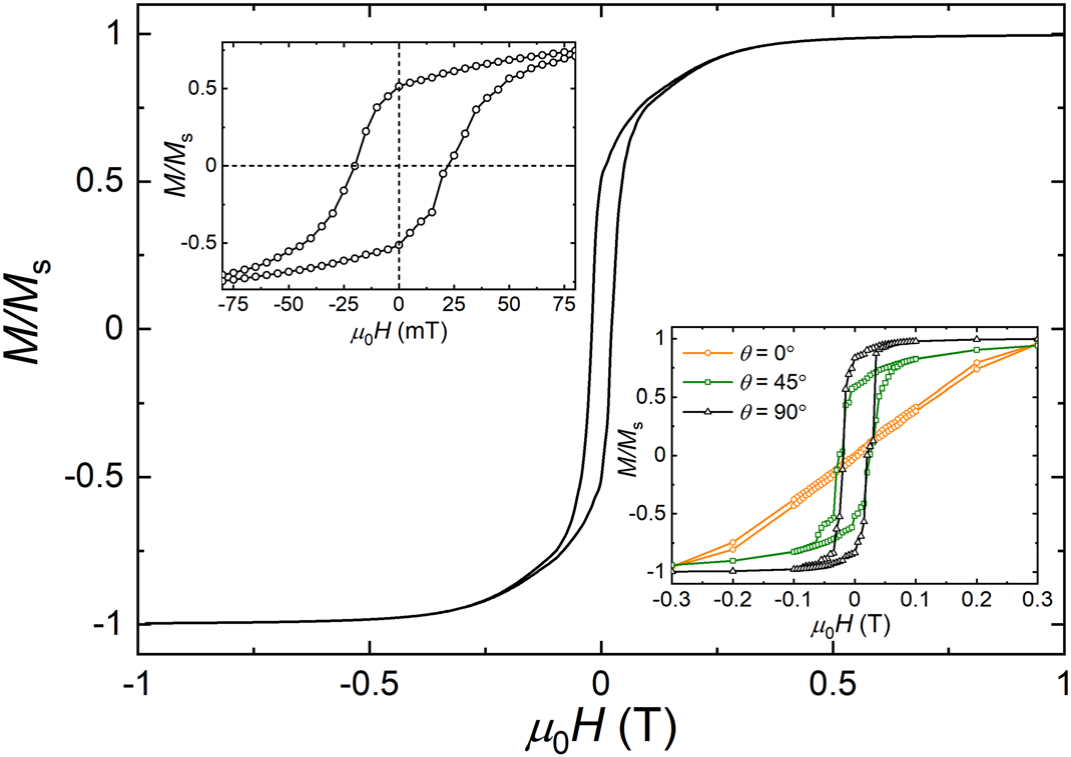
Simulated hysteresis curves. Averaged *M*(*H*) curve for different flake directions with the angle *θ* from the normal of the flake ranging from 0° to 90° with steps of 5°, close-up (left insert) and comparison of selected hysteresis curves yielded with *θ* of 0°, 45°, and 90° (right inset).

**Figure 8:**
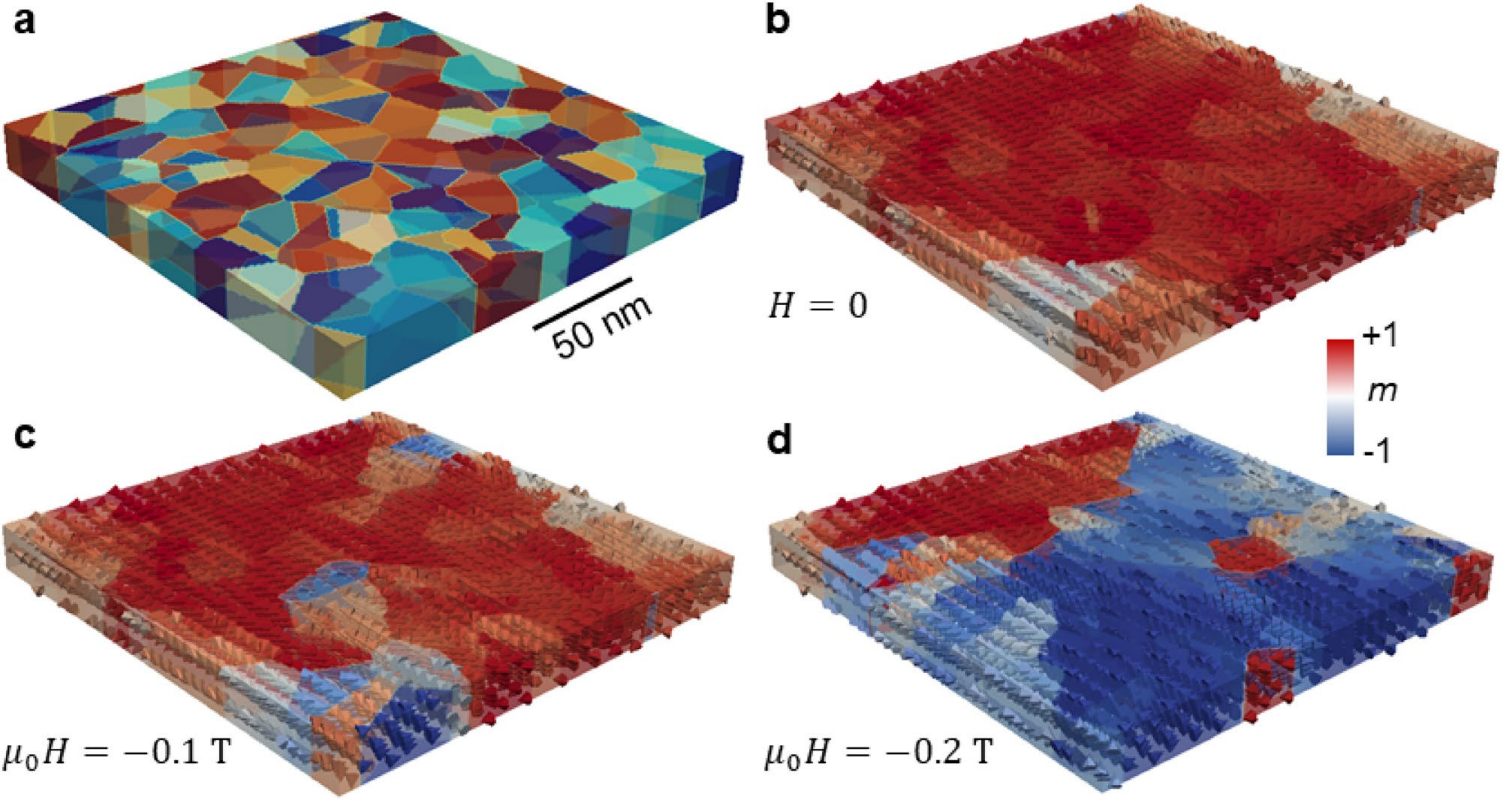
Micromagnetic simulations of a greigite flake. (**a**) exhibits the simulated system that mimics a greigite flake with dimensions of 200 nm × 200 nm × 20 nm consisting of intergrown crystallites with a size of about 20 nm and the colours mirror the different anisotropy vectors. (**b**) Starting from remanence after saturation, (**c**,**d**) the application of an external field generates a CMV configuration.

In order to obtain additional insight to the magnetic configuration of the greigite flakes, we simulated hysteresis curves for different flake directions and performed micromagnetic simulations of a polycrystalline flake generated by means of Voronoi tessellation (see Figs. 7, 8a). The simulation system in Fig. 8a contains blocks which represent intergrown crystallites with different crystallographic orientations and approximate sizes as obtained by XRD and the colours indicate different directions of the anisotropy vector of each block. The average of the convoluted hysteresis curve of a single flake has a coercivity of 20 mT and remanence of *M*_r_ = 0.5 *M*_s_ (Fig. 7), which are in good agreement with the experimental data obtained from the bulk sample (Fig. 2a). The higher coercivity in the measured *M*(*H*) loop is probably due to factors such as anisotropic exchange at the crystallite interfaces and other surface effects that cannot be resolved at this point and are beyond the scope of our micromagnetic model. To account for those contributions, however, atomistic calculations would be required, but the surface/interface parameters for Fe_3_S_4_ are currently unknown.

Importantly, we find that upon the application of an external field, the CMV start to change by overcoming the crystallite to crystallite interfaces (Fig. 8b–d). At any given time, each crystallite and the CMV, which itself contains several crystallites, are in a single-domain state, as it is shown by the experimental and simulated coercivity values. The visualisation of the magnetic state obtained from the simulation and its accordance with the experiments (*M*(*H*), *H*_*c*_) support our interpretation of a dominant texture effect on the magnetization process in the flakes. Even though there are some uncertainties of the material parameters (*M*_s_, *K*, *A*)^24,26,27^ the texture effects would remain prominent under the variation of any of those parameters.

### Concluding comments

Our finding that field-induced changes of the CMV configuration in polycrystalline greigite can generate a reversible instability is of importance in two respects. First, magnetic stability is crucial for the use of greigite in paleomagnetism. Greigite flakes with nano-sized textures exhibit a memory effect in the 10 mT field range as revealed by FMR spectroscopy. This range, however, is magnitudes higher compared to the Earth’s magnetic field of about 0.05 mT, and, therefore, such a weak field induces no modifiable CMV configurations. Hence, the fidelity of greigite as natural remanence carrier in paleomagnetism is not called into doubt by textural effects as long as they are physio-chemically stable. Moreover, the memory effect is temperature-dependent and generates an induced magnetization behaviour unique among magnetic minerals. This can be utilized as a novel trait in order to detect nano-sized, textured greigite as magnetic proxy to reconstruct sulfidic geological systems.

Second, our finding further demonstrates that polycrystalline greigite flakes can be considered as magnetic nano-composites, where SD, inter-grown nano-crystallites form variable CMV configurations. The CMV are manipulable in the range of the coercivity fields and the induced change in the magnetic texture of the flakes can be reset by higher fields. Such memory effect stemmed from the textural architecture provides an invaluable, experimental basis for continuative, fundamental considerations of the influence of intergrown, nanoscopic crystallites on the magnetization of microscopic particles.

## Methods

### Sample preparation

The greigite flakes were synthetized through the phase-controlled colloidal synthesis described by Rhodes et al.^21^. The sample was produced by heating of the mixture of organosulphur precursor dibenzyl disulfide and FeCl_2_ in the presence of oleylamine. In a 25 mL 3-neck round bottom flask, FeCl_2_ (0.50 mmol) and 10 mL of oleylamine were placed under vacuum for 1 h at 60 °C. The temperature was increased to 170 °C and maintained for 1 h under an inert N_2_ atmosphere. In a separate vial, dibenzyl disulfide (1.5 mmol) was dissolved in 5.0 mL of oleylamine and placed under vacuum at room temperature for 5 min followed by backfilling with N_2_ for 15 min. The dibenzyl disulfide solution was then injected into the reaction flask and heated to 220 ℃ for 2 h under N_2_. The reaction solution was then cooled in air to room temperature, and 40 mL if chloroform was added followed by centrifugation for 5 min at 8000 rpm. Particles were further purified by two cycles of suspension with chloroform followed by ethanol (total 20 mL), the centrifugation for 5 min at 4400 rpm. The particles were stored in chloroform.

### Structural characterization

X-ray diffraction analysis was performed on a Rigaku SmartLab X-ray diffractometer equipped with a CuK_α_ radiation source and D/teX Ultra 250 detector, operating at 40 kV and 44 mA. XRD samples were prepared by drop casting a concentrated solution of NPs onto a glass holder. The diffractogram was evaluated with the Bruker AXS TOPAS6.0 program^44^ applying the modified Thompson-Cox-Hastings pseudo-Voigt function^45^. The background was modeled by a polynomial of 6th degree. For the refinement, the structure model by Skinner et al.^1^ was taken. Line shape parameters, the lattice parameter, and global thermal factors for each atomic site were refined. Fractional coordinates and occupation numbers kept untouched. From these fits, the lattice parameters, the MCL size and the stress/strain parameter *ε*_o_ = Δ*d*/*d*, where Δ*d* is the mean deviation of the lattice spacing *d* in per mill, were obtained. The size and the shape of synthesized greigite particles were determined by a transmission electron microscope (FEI Tecnai Osiris S/TEM operating at 200 kV).

### Magnetic and FMR spectroscopic analyses

The magnetic characterization was performed by static and dynamic magnetization experiments at 300 K and at low temperature. A vibrating sample magnetometer (Princeton Measurement Corporation 3900 MicroMag) and Quantum Design Physical Property Measurement System (PPMS) were used to measure hysteresis properties in a 1 T field at 300 K and in a 3 T field down to 10 K, respectively. The coercivity distribution and internal bias fields in a range up to 1 T at 300 K were analysed by FORC diagrams^46^ obtained from 160 curves, increment fields of 1.87 mT, an average time of 100 ms and by using the FORCinel code^47^ with a smoothing factor SF = 8 to compute the first‐order reversal curve distributions. Moreover, in the temperature range between 300 and 10 K, magnetic cycling experiments we performed on demagnetized samples in fields between 10 mT and 1 T using a SQUID magnetometer (MPMS3: Quantum Design) in sweeping modes with rate of 5 K/min in the range between 50 to 300 K and 1 K/min in the range between 50 and 10 K. The analysis of the dynamic magnetization properties comprises the measurement of the amplitude dependence of the *ac* susceptibility at 300 K and ferromagnetic resonance (FMR) spectroscopy. The former measured by a physical property measurement system (PPMS Quantum Design) is used to test the presence of domain walls and the latter to analyze interaction and anisotropy properties^48^. The temperature dependence of the FMR spectra were recorded between 300 and 10 K and the angular dependence at 300 K and 10 K on flakes embedded in paraffin. The resonance field (*H*_re*s*_), defined as the maximum adsorption, and the linewidth (Δ*H*) as peak to peak distance in the first derivative absorption spectra are the key parameters directly obtained from the measurements, and the *g*-value was calculated using the resonance equation ħν = *g*μ_B_*H*_re*s*_ where ħ is the Planck’s constant, ν is the microwave frequency, µ_B_ is the Bohr’s magneton and *g* the effetive *g*-factor^49^. Moreover, the symmetry ratio *A*_*ratio*_ = *H*_high_/*H*_low_ was determined, where the distances between *H*_re*s*_ and the corresponding fields of the peak positions define *H*_low_ and *H*_high_, respectively^39^. The FMR experiments were performed on X-band Bruker ElexSys E500 spectrometer equipped with a goniometer and a cryostat. A sweeping field between 5 and 650 mT, microwave frequency between 9.37 and 9.87 GHz depending on the experiment, a power of 0.2 mW, a modulation amplitude of 0.1 mT were used.

### Micromagnetic simulation

The flake was modelled as platelets consisting of intergrown crystallites with a size of 20 nm. It has a side length of 200 nm and a thickness of 20 nm, and the crystallites were generated by means of Voronoi tessellation. The total energy density of the system, in the continuum approximation, is

$$ \varepsilon = A\left( {\nabla {\mathbf{m}}} \right)^{2} + K\left( {\alpha^{2} \beta^{2} + \beta^{2} \gamma^{2} + \gamma^{2} \alpha^{2} } \right) - \mu_{0} M_{s} {\mathbf{H}} \cdot {\mathbf{m}} - \frac{1}{2}\mu_{0} M_{s} {\mathbf{H}}_{{{\text{dm}}}} \cdot {\mathbf{m}}, $$

where *A* is the exchange stiffness, **m** is the magnetization unit vector ${\mathbf{m}} = {\mathbf{M}}/M_{{\text{s}}}$ with the saturation magnetization $M_{{\text{s}}}$, *K* is the first-order cubic magnetocrystalline anisotropy constant with the directional cosines $\alpha ,\beta ,\gamma$, **H** is the external field and **H**_dm_ is the local demagnetizing field due to dipole–dipole interactions. The material parameters were taken from the literature^24,26,27,50^ to be $A = 2 \times 10^{ - 12}$ J/m, $K = - 1.7 \times 10^{4}$ J/m^3^, and $M_{s} = 2.7 \times 10^{5}$ A/m. These parameters yield an exchange length of $\delta = \sqrt {2A/\mu_{0} M_{{\text{s}}}^{2} } \approx 6.6$ nm. The system was discretized in finite-difference cells with a dimension of 1 nm. The computation of hysteresis curves was performed for different flake directions from normal to in-plane in 5° steps using the software package Mumax3^51^ with the steepest conjugate gradient method, and the magnetization was computed in field-cycles between $\mu_{0} H = \pm 1$ T. The convoluted and averaged curves were taken to predict bulk properties of a flake.
